# Clinical outcomes and pharmacokinetics/pharmacodynamics of intravenous polymyxin B treatment for various site carbapenem-resistant gram-negative bacterial infections: a prospective observational multicenter study

**DOI:** 10.1128/aac.01859-24

**Published:** 2025-03-06

**Authors:** Zhenwei Yu, Huangdu Hu, Xiaofen Liu, Jieqiong Liu, Lingyan Yu, Anqi Wei, Chuanwei Xin, Yongxiong Gan, Shu Lei, Li Zhuang, Yanfei Shen, Xiaoxing Du, Jianping Zhu, Yi Yang, Gang Liang, Feng Guo, Jing Zhang, Yunsong Yu

**Affiliations:** 1 Sir Run Run Shaw Hospital, School of Medicine, Zhejiang University 56660 , Hangzhou, China; 2 Center for General Practice Medicine, Department of Infectious Diseases, Zhejiang Provincial People's Hospital (Affiliated People's Hospital, Hangzhou Medical College) 117839 , Hangzhou, China; 3 Institute of Antibiotics, National Clinical Research Center for Aging and Medicine, Huashan Hospital, Fudan University 12478 , Shanghai, China; 4 Key Laboratory of Clinical Pharmacology of Antibiotics, Shanghai, China; 5 Second Affiliated Hospital, School of Medicine, Zhejiang University 26441 , Hangzhou, China; 6 Department of Intensive Care Unit, Hangzhou Red-Cross Hospital, Hangzhou, China; 7 Tongde Hospital of Zhejiang Province 414282 , Hangzhou, China; 8 The First Affiliated Hospital of Ningbo University 117881 , Ningbo, China; 9 The First Affiliated Hospital of Zhejiang Chinese Medical University 74723 , Hangzhou, China; 10 Shulan (Hangzhou) Hospital, Hangzhou, China; 11 Zhejiang Hospital 584020 , Hangzhou, China; Providence Portland Medical Center, Portland, Oregon, USA

**Keywords:** polymyxin B, mortality, acute kidney injury, pharmacokinetic/pharmacodynamic, AUC

## Abstract

Polymyxin B, a last resort for carbapenem-resistant gram-negative bacteria (CRGNB) infections, has infection site-specific pharmacokinetic/pharmacodynamic (PK/PD) properties. However, there is little clinical evidence to support optimal exposures of polymyxin B for different site infections. We performed a prospective, observational, multicenter study to evaluate the clinical outcomes and PK/PD of intravenous polymyxin B treatment for various site CRGNB infections. The main clinical outcomes were 14-day all-cause mortality and nephrotoxicity, and the secondary outcomes were 28-day mortality and clinical response. The area under curves (AUCs) of polymyxin B were determined, and their associations with clinical outcomes were analyzed by stratification based on the infection site. A total of 312 patients were ultimately enrolled from 10 research centers. The overall 14-day mortality was 29.5%, and those of patients with lower respiratory tract infection (LRTI), intra-abdominal infection (IAI), and bloodstream infection (BSI) were 32.3%, 19.7%, and 30.3%, respectively. The 28-day mortality rate was 38.1%, while LRTI patients had the highest mortality (41.4%) and IAI patients lowest (34.8%). The clinical response rate was 46.2%, which was similar among the subgroups. The overall AKI rate was 60.9%. An AUC greater than 50 mg∙h/L was related to lower mortality in IAI patients but not in LRTI patients, which led to a lower but not significant difference in the overall analysis. The AUC of polymyxin B was an independent risk factor for 14-day mortality in IAI patients, and the cutoff value was 76 mg∙h/L. The results would be helpful for personalized dosing and monitoring of polymyxin B.

**CLINICAL TRIALS:**

This study is registered with the Chinese Clinical Trial Registry as ChiCTR2200056667.

## INTRODUCTION

The emergence of multidrug-resistant pathogens has become a major challenge for healthcare providers (1). Infection caused by carbapenem-resistant gram-negative bacteria (CRGNB), mainly carbapenem-resistant *Klebsiella pneumoniae* (CRKP), carbapenem-resistant *Pseudomonas aeruginosa* (CRPA), and carbapenem-resistant *Acinetobacter baumannii* (CRAB), leads to increased mortality, longer hospital stays, and additional costs (2–5). Unfortunately, drugs that are susceptible to CRGNB are few in number (6).

Polymyxin, a fast bacterial-killing agent once abandoned due to its severe neurotoxicity and nephrotoxicity, had been brought back into the field of clinical application (7). It has good *in vitro* activity against CRGNBs and is considered one of the “last-resort” of CRGNB infection (8). Although novel beta-lactam/beta-lactamase inhibitors against CRGNB are available in recent years, polymyxins still play an important role, especially in developing countries (9). There are two main forms of polymyxins available on the market: colistin methanesulfonate (CMS) and polymyxin B sulfate. Although their molecular structures are similar, they involve distinct *in vivo* processes (10). Polymyxin B is administered in its active form and is considered to have superior pharmacological properties to CMS (11).

Polymyxin B has a narrow therapeutic window as low exposure leads to treatment failure and heterogeneous resistance, but high exposure can cause significant neurotoxicity and nephrotoxicity (12–14). It should be dosed under the guidance of the pharmacokinetic/pharmacodynamic (PK/PD) principle, but there are limited data to suggest an optimal exposure of polymyxin B to balance the efficacy and safety. A preclinical PK/PD study revealed that the free-drug area under the curve over the minimum inhibitory concentration (*f*AUC/MIC) was the PK/PD index associated with bacterial killing, and its value should be above 3.72–28 for 2 log kill of KP in thigh infection models (15). Thus, the 2019 international consensus guideline recommended an AUC target of 50–100 mg∙h/L without sufficient clinical efficacy data (16). However, polymyxin B has site-specific PD properties. Studies in animal models have suggested that a much higher PK/PD target for lung infection is needed and that bacterial stasis cannot be achieved for some strains (15). Polymyxin B treatment also resulted in poorer clinical outcomes in patients with lower respiratory tract infection (LRTI) than in those with other site infections. Recent PK/PD studies including patients with various infection sites have suggested optimal AUC thresholds, but the results are inconclusive (17). Considering the viability of PKs and the uncertainty in PK/PD, it remains a challenge to dose polymyxin B in clinical practice (18). The Prato polymyxin consensus claimed that larger pharmacokinetic/pharmacodynamic and clinical studies of polymyxin B are urgently needed to develop improved dosing strategies with this drug (19).

Thus, we designed a prospective observational multicenter study to assess the clinical outcomes and PK/PD of intravenous polymyxin B treatment for various site CRGNB infection and to determine the optimal exposure range of polymyxin B, which would be helpful in the future clinical application of this drug.

## RESULTS

### Patient inclusion and characteristics and distribution of the AUC

**Fig 1:**
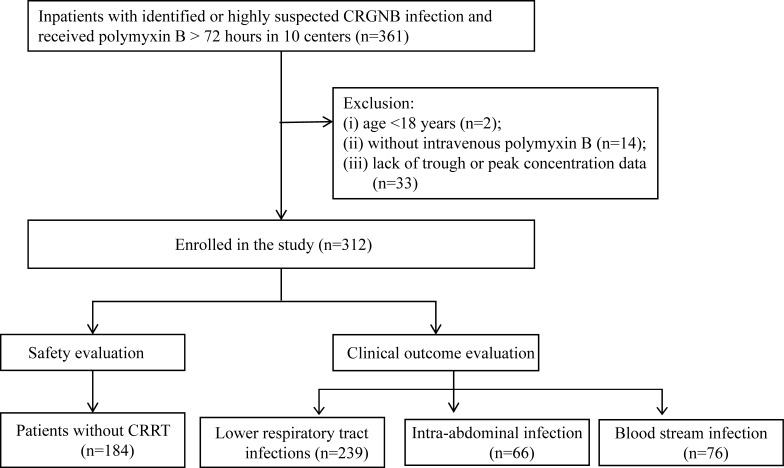
Flowchart of patient enrollment. CRGNB, carbapenem-resistant gram-negative bacteria; CRRT, continuous renal replacement treatment.

As shown in Fig. 1, a total of 312 patients were ultimately enrolled from 10 research centers. All patients were eligible for clinical outcome evaluation, and 184 of those patients were eligible for nephrotoxicity analysis. The detailed patient characteristics are shown in Table 1. Most of the patients were old and admitted to the ICU. The main infection type was lower respiratory tract infection (LRTI), and some patients had multiple infection sites.

**TABLE 1:** Demographic characteristics of the included patients^*a*^

Variable	Total (*n* = 312)	Subgroups			
		Non-CRRT (*n* = 184)	LRTIs (*n* = 239)	IAIs (*n* = 66)	BSIs (*n* = 76)
Sex, *n* (%)					
Male	221 (70.8%)	128 (69.6%)	178 (74.5%)	41 (62.1%)	53 (69.7%)
Female	91 (29.2%)	56 (30.4%)	61 (25.5%)	25 (37.9%)	23 (30.3%)
Age (years)	69.0 (58.0, 80.0)	69.0 (58.0, 79.0)	72.0 (61.0, 82.0)	59.5 (51.0, 69.0)	68.0 (58.0, 77.0)
Weight (kg)	60.5 (54.6, 70.0)	60.5 (54.1, 70.0)	60.0 (54.0, 70.0)	60.0 (55.0, 70.0)	65.0 (55.5, 70.0)
BMI (kg/m^2^)	22.1 (19.8, 24.8)	22.0 (19.6, 24.8)	22.0 (19.6, 24.5)	22.9 (20.6, 25.1)	23.1 (20.8, 26.0)
Days of hospital stay	45.0 (27.0, 76.0)	53.5 (30.0, 85.0)	43.0 (27.0, 70.0)	61.5 (38.0, 99.0)	43.0 (27.0, 80.0)
Comorbidities, *n* (%)					
Dementia	14 (4.50%)	7 (3.80%)	13 (5.40%)	0 (0.00%)	3 (3.90%)
Diabetes	85 (27.2%)	50 (27.2%)	69 (28.9%)	8 (12.1%)	24 (31.6%)
Chronic kidney disease	35 (11.2%)	10 (5.40%)	30 (12.6%)	5 (7.60%)	9 (11.8%)
Malignancy	75 (24.0%)	52 (28.3%)	53 (22.2%)	13 (19.7%)	16 (21.1%)
Heart failure	40 (12.8%)	20 (10.9%)	37 (15.5%)	1 (1.50%)	7 (9.20%)
COPD	30 (9.60%)	20 (10.9%)	28 (11.7%)	0 (0.00%)	4 (5.30%)
Immunodeficiency	20 (6.40%)	6 (3.30%)	18 (7.50%)	3 (4.50%)	7 (9.20%)
Hypertension	145 (46.5%)	76 (41.3%)	114 (47.7%)	21 (31.8%)	36 (47.4%)
Hyperlipidemia	9 (2.90%)	6 (3.30%)	5 (2.10%)	5 (7.60%)	1 (1.30%)
Acute pancreatitis	37 (11.9%)	21 (11.4%)	15 (6.30%)	31 (47.0%)	8 (10.5%)
COVID-19	38 (12.2%)	21 (11.4%)	34 (14.2%)	5 (7.60%)	9 (11.8%)
ICU admission	290 (92.9%)	162 (88.0%)	230 (96.2%)	65 (98.5%)	68 (89.5%)
Days of ICU stay	30.0 (19.0, 53.0)	35.5 (18.0, 60.0)	35.0 (19.0, 56.0)	37.0 (22.0, 51.0)	33.0 (20.0, 54.0)
SOFA score	8.00 (5.00, 12.0)	6.00 (4.00, 9.00)	9.00 (6.00, 12.0)	8.00 (4.00, 12.0)	8.00 (5.00, 12.0)
APACHE II score	24.0 (17.0, 29.0)	21.0 (15.0, 27.0)	24.5 (18.0, 30.0)	24.5 (15.0, 28.0)	22.0 (17.0, 29.0)
Severely ill	160 (51.3%)	70 (38.0%)	132 (55.2%)	32 (48.5%)	38 (50.0%)
Mechanical ventilation	229 (73.4%)	128 (69.6%)	190 (79.5%)	42 (63.6%)	51 (67.1%)
Surgical removal of infection	43 (13.8%)	25 (13.6%)	23 (9.60%)	27 (40.9%)	12 (15.8%)
Infected site, *n* (%)					
LRTIs	239 (76.6%)	137 (74.5%)	239 (100%)	28 (42.4%)	46 (60.5%)
IAIs	66 (21.2%)	32 (17.4%)	28 (11.7%)	66 (100%)	11 (14.5%)
BSIs	76 (24.4%)	42 (22.8%)	46 (19.2%)	11 (16.7%)	76 (100%)
Other or undefined	36 (11.5%)	21 (11.4%)	23 (9.60%)	7 (10.6%)	10 (13.2%)
Pathogen, *n* (%)					
CRKP	86 (27.6%)	51 (27.7%)	55 (23.0%)	34 (51.5%)	27 (35.5%)
CRPA	65 (20.8%)	37 (20.1%)	52 (21.8%)	15 (22.7%)	10 (13.2%)
CRAB	141 (45.2%)	73 (39.7%)	128 (53.6%)	30 (45.5%)	31 (40.8%)
Others	137 (43.9%)	84 (45.7%)	107 (44.8%)	28 (42.4%)	47 (61.8%)
CRRT	128 (41.0%)	0 (0.00%)	102 (42.7%)	34 (51.5%)	34 (44.7%)
ECMO	23 (7.40%)	7 (3.80%)	23 (9.60%)	1 (1.50%)	6 (7.90%)
Daily dose (mg/d)	150 (100, 150)	150 (100, 150)	150 (100, 150)	150 (150, 150)	150 (100, 150)
Dose/weight (mg/kg/12 hours)	1.05 (0.833, 1.25)	1.00 (0.833, 1.25)	1.00 (0.833, 1.25)	1.14 (0.995, 1.36)	1.05 (0.833, 1.15)
Days of therapy	10.0 (7.00, 16.0)	10.0 (7.00, 16.0)	10.0 (7.00, 15.0)	14.0 (7.00, 22.0)	9.50 (7.00, 15.0)
Aerosol inhalation	45 (14.4%)	23 (12.5%)	41 (17.2%)	2 (3.00%)	14 (18.4%)
Concomitant drugs, *n* (%)					
Vasoactive drugs	151 (48.4%)	77 (41.8%)	123 (51.5%)	26 (39.4%)	38 (50.0%)
Nephrotoxic drugs	101 (32.4%)	58 (31.5%)	74 (31.0%)	32 (48.5%)	19 (25.0%)
Tigecycline	103 (33.0%)	59 (32.1%)	73 (30.5%)	25 (37.9%)	32 (42.1%)
CAZ-AVI	43 (13.8%)	18 (9.80%)	32 (13.4%)	11 (16.7%)	13 (17.1%)
Carbapenem	60 (19.2%)	30 (16.3%)	42 (17.6%)	17 (25.8%)	17 (22.4%)
BLBLI	105 (33.7%)	64 (34.8%)	88 (36.8%)	19 (28.8%)	20 (26.3%)
Aminoglycosides	10 (3.20%)	4 (2.20%)	8 (3.30%)	2 (3.00%)	1 (1.30%)
Others	49 (15.7%)	29 (15.8%)	37 (15.5%)	14 (21.2%)	12 (15.8%)
None	69 (22.1%)	44 (23.9%)	56 (23.4%)	11 (16.7%)	18 (23.7%)
AUC_ss,24 h_ (mg·h/L)	76.4 (55.0, 107)	79.9 (59.6, 114)	76.5 (56.1, 106)	72.9 (52.8, 98.4)	79.7 (58.6, 109)
AUC ≥50 mg·h/L	251 (80.4%)	154 (83.7%)	194 (81.2%)	52 (78.8%)	67 (88.2%)
Laboratory data					
CRP (μg/L)	124 (74.6, 187)	111 (67.0, 158)	121 (69.5, 185)	134 (91.4, 187)	125 (83.7, 179)
PCT (ng/mL)	1.38 (0.476, 5.61)	0.830 (0.281, 2.62)	1.22 (0.422, 4.66)	1.92 (0.800, 13.1)	2.74 (0.905, 14.1)
RBC (10^12^ /L)	2.50 (2.18, 2.92)	2.65 (2.28, 3.01)	2.55 (2.21, 2.95)	2.41 (2.00, 2.63)	2.57 (2.08, 2.96)
WBC (10^9^ /L)	10.1 (6.05, 15.0)	9.65 (6.48, 14.2)	10.3 (6.30, 14.6)	11.7 (6.19, 18.6)	10.6 (5.40, 16.4)
Neutrophil (10^9^ /L)	87.1 (78.7, 92.1)	86.5 (76.0, 91.4)	87.4 (79.0, 92.5)	89.0 (81.4, 91.8)	88.0 (74.9, 93.1)
SCr (μmol/L)	76.8 (54.0, 120)	63.5 (46.0, 95.6)	78.0 (54.0, 114)	75.0 (58.0, 122)	89.0 (60.8, 146)
BUN (mmol/L)	11.0 (6.50, 16.8)	8.97 (5.50, 13.6)	11.7 (7.20, 17.2)	9.68 (5.70, 15.3)	13.0 (6.99, 20.3)
ALB (g/L)	29.3 (26.3, 31.6)	29.3 (26.2, 31.6)	29.3 (26.6, 31.7)	29.5 (26.6, 32.5)	29.3 (25.9, 31.9)
TP (g/L)	54.6 (49.9, 60.1)	55.2 (50.1, 60.0)	54.6 (50.6, 60.1)	54.3 (49.3, 58.2)	54.9 (49.3, 60.7)
ALT (U/L)	30.0 (15.0, 60.0)	29.0 (15.0, 55.0)	30.0 (15.0, 57.3)	25.5 (14.8, 46.5)	32.0 (14.3, 73.8)
AST (U/L)	37.0 (23.5, 65.5)	34.0 (22.0, 56.0)	36.0 (24.0, 65.0)	38.5 (23.0, 56.8)	42.0 (24.0, 69.5)
ALP (U/L)	116 (80.0, 171)	102 (76.0, 162)	107 (77.5, 160)	143 (102, 203)	104 (84.0, 175)
TBIL (μmol/L)	16.1 (9.48, 39.6)	12.7 (8.70, 22.5)	15.3 (9.10, 36.4)	50.8 (16.8, 139)	18.0 (8.75, 47.9)

^*a*^ OR, odds ratio; CRRT, continuous renal replacement therapy; LRTIs, lower respiratory tract infections; IAIs, intra-abdominal infections; BSIs, bloodstream infections; BMI, body mass index; COPD, chronic obstructive pulmonary disease; ICU, intensive care unit; SOFA, sequential organ failure assessment; APACHE, acute physiology and chronic health evaluation; CRKP, carbapenem-resistant *Klebsiella pneumoniae*; CRPA, carbapenem-resistant *Pseudomonas aeruginosa*; CRAB, carbapenem-resistant *Acinetobacter baumannii*; ECMO, extracorporeal membrane oxygenation; CAZ-AVI, ceftazidime-avibactam; BLBLI, beta-lactam/beta-lactamase inhibitor combinations; AUC_ss,24 h_, area under the concentration-to-time curve across 24 hours at steady state; CRP; C-reactive protein; PCT, procalcitonin; RBC, red blood cell; WBC, white blood cell; SCr, serum creatinine; BUN, blood urea nitrogen; ALB, serum albumin; TP; total protein; ALT, alanine aminotransferase; AST, aspartate aminotransferase; ALP, alkaline phosphatase; TBIL, total bilirubin.

### Clinical outcomes

**Fig 2:**
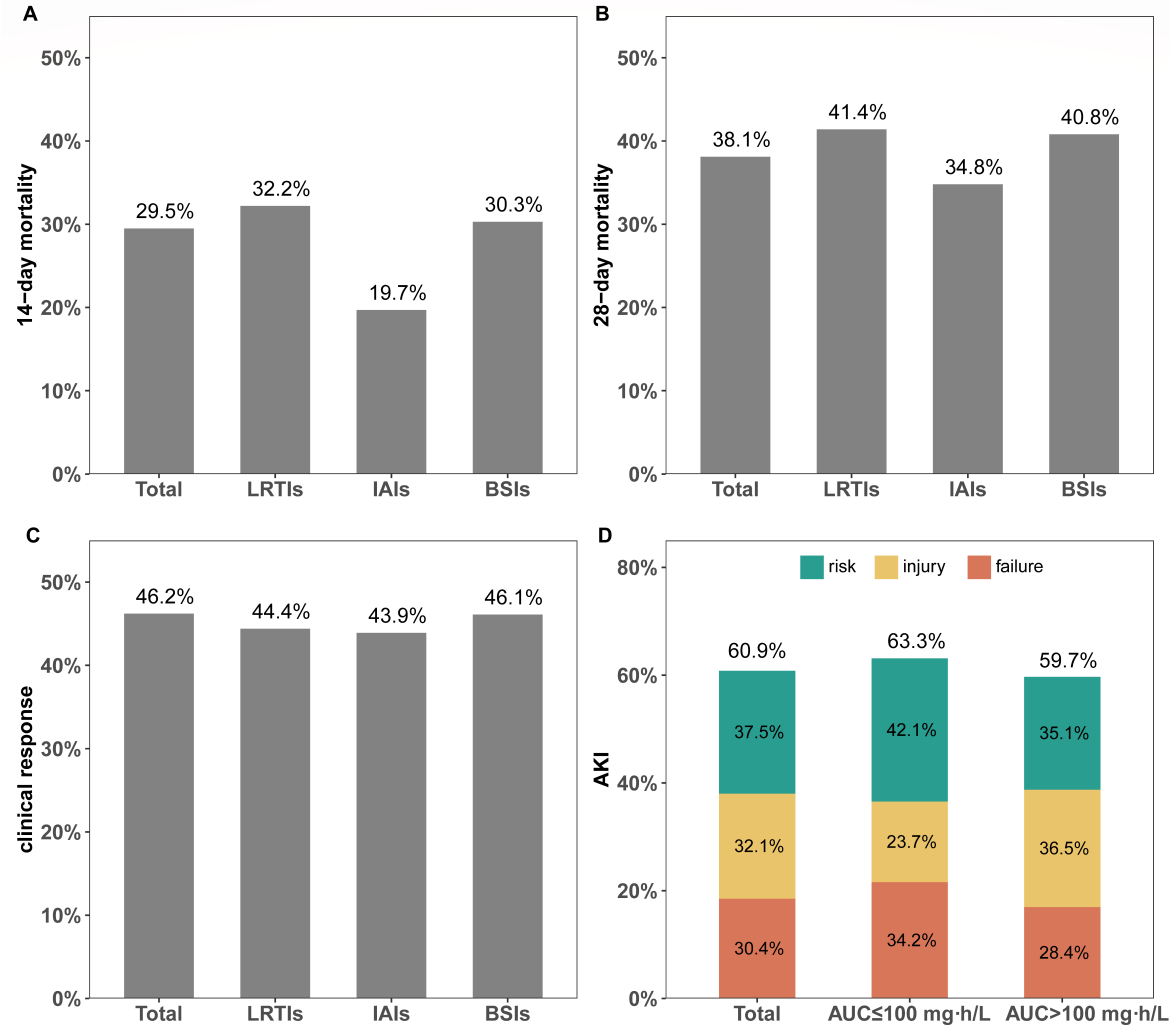
Clinical outcome of intravenous polymyxin B treatment for patients with CRGNB infection. (A) 14-day mortality; (B) 28-day mortality; (C) clinical response rate; (D) AKI rate. LRTI, lower respiratory tract infection; IAI, intra-abdominal infection; BSI, bloodstream infection; AKI, acute kidney injury; AUC, area under the curve of polymyxin B.

The overall 14-day mortality was 29.5%, while patients with LRTI and bloodstream infections (BSI) had higher mortality rates (Fig. 2; Table 2). The 28-day mortality rate was 38.1%, while LRTI patients had the highest mortality (41.4%), and intra-abdominal infection (IAI) patients had the lowest (34.8%). However, the clinical response rate was 46.2%, which was similar among the subgroups. Among the patients eligible for nephrotoxicity analysis, the overall acute kidney injury (AKI) rate was 60.9%.

**TABLE 2:** Clinical outcomes of the included patients^*a*^

Variable	Total (*n* = 312)	Subgroups			
		Non-CRRT (*n* = 184)	LRTIs (*n* = 239)	IAIs (*n* = 66)	BSIs (*n* = 76)
Outcomes					
Clinical response	144 (46.2%)	99 (53.8%)	106 (44.4%)	29 (43.9%)	35 (46.1%)
14-day all-cause mortality	92 (29.5%)	40 (21.7%)	77 (32.2%)	13 (19.7%)	23 (30.3%)
28-day all-cause mortality	119 (38.1%)	51 (27.7%)	99 (41.4%)	23 (34.8%)	31 (40.8%)
Occurrence of AKI					
No	72 (39.1%)	72 (39.1%)	51 (37.2%)	10 (31.2%)	15 (34.9%)
Yes	112 (60.9%)	112 (60.9%)	86 (62.8%)	22 (68.8%)	28 (65.1%)
AKI stage					
Risk	42 (37.5%)	42 (37.5%)	35 (40.7%)	5 (22.7%)	11 (39.3%)
Injury	36 (32.1%)	36 (32.1%)	26 (30.2%)	11 (50.0%)	9 (32.1%)
Failure	34 (30.4%)	34 (30.4%)	25 (29.1%)	6 (27.3%)	8 (28.6%)

^*a*^ CRRT, continuous renal replacement therapy; LRTIs, lower respiratory tract infections; IAIs, intra-abdominal infections; BSIs, bloodstream infections; AKI, acute kidney injury.

### PK/PD analysis

**Fig 3:**
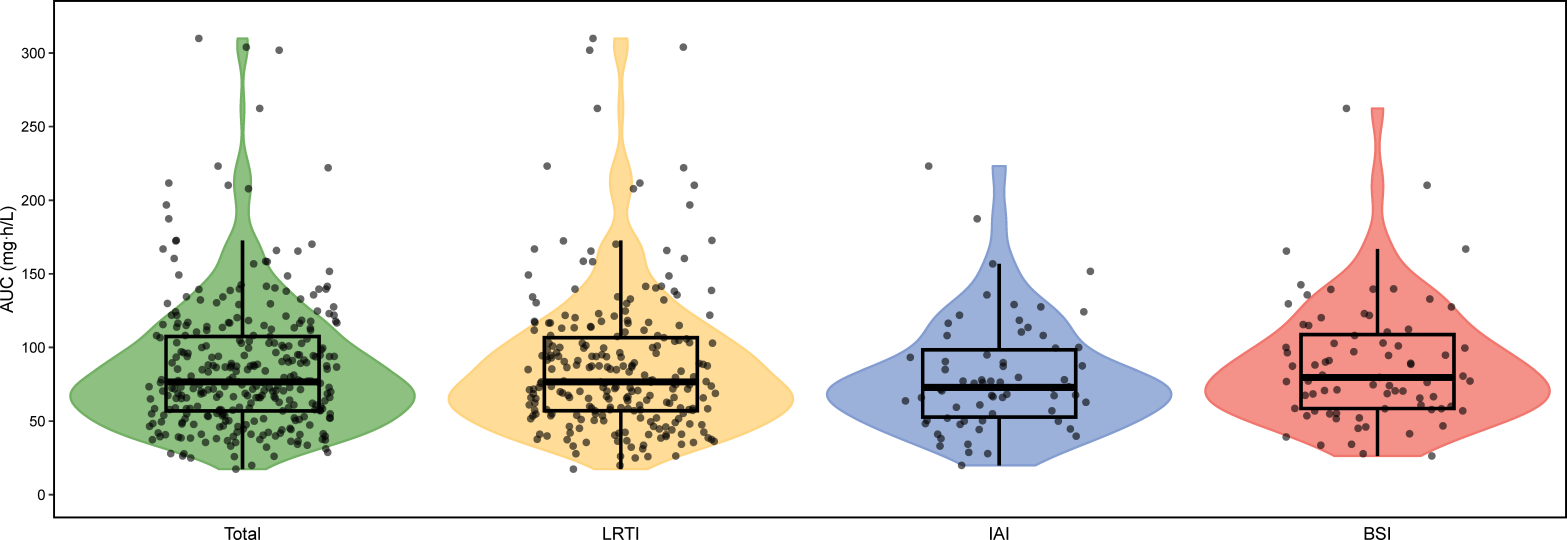
Distribution of polymyxin B steady-state 24-hour area under the curve in patients. LRTI, lower respiratory tract infection; IAI, intra-abdominal infection; BSI, bloodstream infection.

**Fig 4:**
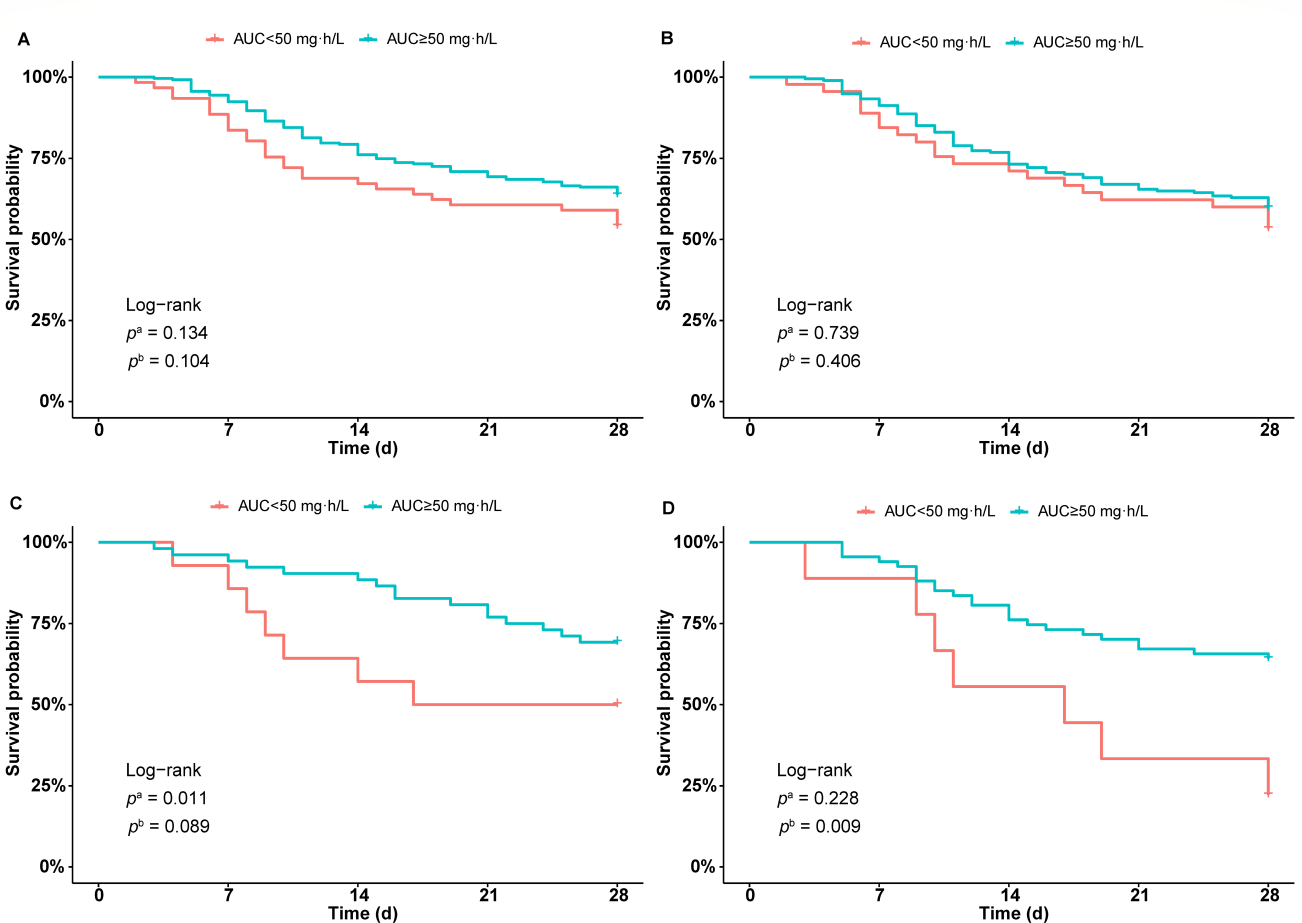
Survival analysis of patients with different levels of systemic polymyxin B exposure. (**A**) Overall patients; (**B**) patients with lower respiratory tract infection; (**C**) intra-abdominal infection; (**D**) bloodstream infection. AUC, area under the curve of polymyxin B. *p*^a^ and *p*^b^ indicate significant differences in 14-day and 28-day survival between the two groups, respectively.

The polymyxin B AUC distributions in whole patients and subgroups are shown in Fig. 3. Kaplan–Meier analysis was performed to test the differences in mortality between patients with different polymyxin B exposures. When we stratified the patients according to the AUC 50 mg∙h/L threshold, the overall survival curve revealed that patients with AUCs over 50 mg∙h/L tended to have lower mortality, but the differences were not significant (Fig. 4A). However, the curves were similar in patients with LRTI (Fig. 4B). IAI patients with an AUC over 50 mg∙h/L had significantly lower 14-day mortality (Fig. 4C). However, BSI patients had lower 28-day mortality if the AUC was greater than 50 mg∙h/L (Fig. 4D).

The multivariate logistic regression identified several independent risk factors for overall 14-day mortality, but the AUC did not (Table 3). However, the AUC and use of CRRT were independent risk factors for 14-day mortality in IAI patients (Table 4). In patients with LRTI and BSI, the AUC was not associated with 14-day mortality (Tables S1 and S2). The results of multivariate logistic regression for secondary outcomes and other infection types are shown in Tables S3 to S11. Only in patients with IAI was the AUC associated with 28-day mortality (Table S8).

**TABLE 3:** Univariate and multivariate logistic regression analyses for 14-day mortality in all patients^*a*^

	Survival (*n* = 220)	No survival (*n* = 92)	Univariate logistic regression	Multivariate logistic regression	
			*P*-value	OR (95% CI)	*P*-value
Sex, *n* (%)					
Male	156 (70.9%)	65 (70.7%)	0.964		
Female	64 (29.1%)	27 (29.3%)			
Age (years)	68.0 (57.0, 78.0)	73.5 (61.0, 82.0)	0.063		0.660
Weight (kg)	60.5 (55.0, 70.0)	61.5 (51.4, 70.0)	0.407		
BMI (kg/m^2^)	22.0 (20.0, 24.8)	22.5 (19.5, 25.8)	0.277		
Comorbidities, *n* (%)					
Dementia	10 (4.50%)	4 (4.30%)	0.939		
Diabetes	53 (24.1%)	32 (34.8%)	0.054		0.584
CKD	23 (10.5%)	12 (13.0%)	0.510		
Malignancy	55 (25.0%)	20 (21.7%)	0.539		
Heart failure	26 (11.8%)	14 (15.2%)	0.414		
COPD	23 (10.5%)	7 (7.60%)	0.439		
Immunodeficiency	17 (7.70%)	3 (3.30%)	0.154		0.201
Hypertension	101 (45.9%)	44 (47.8%)	0.757		
Hyperlipidemia	6 (2.70%)	3 (3.30%)	0.798		
Acute pancreatitis	30 (13.6%)	7 (7.60%)	0.139		
COVID-19	17 (7.70%)	21 (22.8%)	*P* < 0.001	3.90 (1.70–8.97)	0.001
ICU admission	199 (90.5%)	91 (98.9%)	0.028		0.480
Severely ill	105 (47.7%)	55 (59.8%)	0.053		
Infected site, *n* (%)					
LRTIs	162 (73.6%)	77 (83.7%)	0.058		
IAIs	53 (24.1%)	13 (14.1%)	0.052		0.185
BSIs	53 (24.1%)	23 (25.0%)	0.865		
Other or undefined	24 (10.9%)	12 (13.0%)	0.591		
Pathogen, *n* (%)					
CRKP	68 (30.9%)	18 (19.6%)	0.043		0.368
CRPA	47 (21.4%)	18 (19.6%)	0.721		
CRAB	90 (40.9%)	51 (55.4%)	0.019		0.302
others	104 (47.3%)	33 (35.9%)	0.065		
CRRT	76 (34.5%)	52 (56.5%)	*P* < 0.001	2.65 (1.41–4.99)	0.002
ECMO	16 (7.30%)	7 (7.60%)	0.917		
Daily dose (mg/d)	150 (100, 150)	150 (100, 150)	0.630		
Dose/weight (mg/kg/12 hours)	1.05 (0.833, 1.25)	1.06 (0.833, 1.28)	0.909		
Aerosol inhalation	34 (15.5%)	11 (12.0%)	0.424		
Concomitant drugs, *n* (%)					
Tigecycline	70 (31.8%)	33 (35.9%)	0.488		
CAZ-AVI	36 (16.4%)	7 (7.60%)	0.046	0.179 (0.057–0.562)	0.003
Carbapenem	42 (19.1%)	18 (19.6%)	0.923		
BLBLI	73 (33.2%)	32 (34.8%)	0.785		
Aminoglycosides	5 (2.30%)	5 (5.40%)	0.161	6.27 (1.42–27.7)	0.016
Other	40 (18.2%)	9 (9.80%)	0.067		
None	51 (23.2%)	18 (19.6%)	0.483		
AUC_ss,24 h_ (mg·h/L)	76.6 (57.3, 108)	76.3 (51.8, 103)	0.976		
AUC ≥50 mg·h/L	181 (82.3%)	70 (76.1%)	0.211		
Laboratory data					
CRP (μg/L)	121 (73.1, 184)	131 (77.5, 193)	0.516		
PCT (ng/mL)	1.19 (0.402, 5.91)	1.88 (0.613, 5.44)	0.503		
RBC (10^12^ /L)	2.51 (2.19, 2.95)	2.48 (2.16, 2.89)	0.366		
WBC (10^9^ /L)	9.60 (5.90, 14.2)	11.4 (6.60, 17.2)	0.024		0.063
Neutrophil (10^9^ /L)	85.8 (76.6, 91.5)	90.0 (83.5, 93.4)	0.046		
SCr (μmol/L)	72.0 (53.0, 110)	96.3 (61.5, 152)	0.018		
BUN (mmol/L)	9.54 (6.18, 15.1)	13.6 (8.76, 22.8)	*P* < 0.001	1.04 (1.02–1.08)	0.003
ALB (g/L)	29.5 (26.9, 32.0)	28.0 (25.7, 30.7)	0.006		0.076
TP (g/L)	55.6 (51.2, 60.8)	53.2 (47.7, 57.9)	0.014		0.874
ALT (U/L)	29.0 (15.0, 60.0)	31.0 (14.0, 58.8)	0.412		
AST (U/L)	34.0 (21.0, 64.0)	40.0 (28.0, 69.5)	0.585		
ALP (U/L)	120 (82.3, 177)	104 (69.0, 151)	0.124		0.120
TBIL (μmol/L)	15.4 (9.83, 37.9)	17.9 (9.15, 40.2)	0.390		
AKI	88 (61.1%)	24 (60.0%)	0.899		

^*a*^ OR, odds ratio; BMI, body mass index; CKD, chronic kidney disease; COPD, chronic obstructive pulmonary disease; ICU, intensive care unit; LRTIs, lower respiratory tract infections; IAIs, intra-abdominal infections; BSIs, bloodstream infections; CRKP, carbapenem-resistant *Klebsiella pneumoniae*; CRPA, carbapenem-resistant *Pseudomonas aeruginosa*; CRAB, carbapenem-resistant *Acinetobacter baumannii*; CRRT, continuous renal replacement therapy; ECMO, extracorporeal membrane oxygenation; CAZ-AVI, ceftazidime/avibactam; BLBLI, beta-lactam-beta-lactamase inhibitor combinations; AUC_ss,24 h_, area under the concentration-to-time curve across 24 hours at steady state; CRP; C-reactive protein; PCT, procalcitonin; RBC, red blood cell; WBC, white blood cell; SCr, serum creatinine; BUN, blood urea nitrogen; ALB, serum albumin; TP; total protein; ALT, alanine aminotransferase; AST, aspartate aminotransferase; ALP, alkaline phosphatase; TBIL, total bilirubin; AKI, acute kidney injury.

**TABLE 4:** Univariate and multivariate logistic regression analyses of 14-day mortality in intra-abdominal infection patients^*a*^

	Survival (*n* = 53)	No survival (*n* = 13)	Univariate logistic regression	Multivariate logistic regression	
			*P*-value	OR (95% CI)	*P*-value
Sex, *n* (%)					
Male	33 (62.3%)	8 (61.5%)	0.961		
Female	20 (37.7%)	5 (38.5%)			
Age (years)	59.0 (53.0, 69.0)	63.0 (49.0, 71.0)	0.923		
Weight (kg)	63.0 (55.0, 70.0)	60.0 (55.0, 70.0)	0.137		0.826
BMI (kg/m^2^)	22.8 (20.6, 25.2)	23.3 (21.1, 24.8)	0.123		
Comorbidities, *n* (%)					
Diabetes	6 (11.3%)	2 (15.4%)	0.689		
CKD	4 (7.50%)	1 (7.70%)	0.986		
Malignancy	11 (20.8%)	2 (15.4%)	0.664		
Heart failure	1 (1.90%)	0 (0.00%)	0.992		
Immunodeficiency	3 (5.70%)	0 (0.00%)	0.994		
Hypertension	17 (32.1%)	4 (30.8%)	0.928		
Hyperlipidemia	3 (5.70%)	2 (15.4%)	0.254		
Acute pancreatitis	26 (49.1%)	5 (38.5%)	0.495		
COVID-19	5 (9.40%)	0 (0.00%)	0.993		
ICU admission	52 (98.1%)	13 (100%)	0.992		
Severely ill	22 (41.5%)	10 (76.9%)	0.030		
Surgical removal of infection	25 (47.2%)	2 (15.4%)	0.027	0.004 (0.001–0.381)	0.017
Infected site, *n* (%)					
LRTIs	21 (39.6%)	7 (53.8%)	0.356		
BSIs	9 (17.0%)	2 (15.4%)	0.890		
Other or undefined	5 (9.40%)	2 (15.4%)	0.536		
Pathogen, *n* (%)					
CRKP	26 (49.1%)	8 (61.5%)	0.422		
CRPA	10 (18.9%)	5 (38.5%)	0.140		0.513
CRAB	22 (41.5%)	8 (61.5%)	0.200		
Others	25 (47.2%)	3 (23.1%)	0.126		
CRRT	22 (41.5%)	12 (92.3%)	0.009		0.232
ECMO	0 (0.00%)	1 (7.70%)	0.991		
Daily dose (mg/d)	150 (100, 150)	150 (150, 150)	0.197		
Dose/weight (mg/kg/12 hours)	1.15 (1.00, 1.36)	1.07 (0.833, 1.32)	0.338		
Aerosol inhalation	2 (3.80%)	0 (0.00%)	0.993		
Concomitant drugs, *n* (%)					
Tigecycline	19 (35.8%)	6 (46.2%)	0.494		
CAZ-AVI	10 (18.9%)	1 (7.70%)	0.350		
Carbapenem	12 (22.6%)	5 (38.5%)	0.249		
BLBLI	17 (32.1%)	2 (15.4%)	0.246		
Aminoglycosides	2 (3.80%)	0 (0.00%)	0.993		
Others	10 (18.9%)	4 (30.8%)	0.352		
None	10 (18.9%)	1 (7.70%)	0.350		
AUC_ss,24 h_ (mg·h/L)	77.2 (62.8, 108)	50.5 (41.1, 66.6)	0.014	0.936 (0.886–0.989)	0.018
AUC ≥50 mg·h/L	45 (84.9%)	7 (53.8%)	0.020		
Laboratory data					
CRP (μg/L)	130 (87.2, 186)	147 (91.6, 217)	0.409		
PCT (ng/mL)	1.89 (0.800, 10.5)	2.62 (1.34, 14.0)	0.651		
RBC (10^12^ /L)	2.29 (1.98, 2.67)	2.44 (2.21, 2.48)	0.698		
WBC (10^9^ /L)	11.2 (6.12, 16.4)	17.3 (8.60, 23.7)	0.092	1.144 (1.017–1.287)	0.026
Neutrophil (10^9^ /L)	89.0 (81.3, 92.6)	89.0 (82.6, 90.3)	0.934		
SCr (μmol/L)	73.0 (58.0, 116)	89.0 (62.0, 136)	0.816		
BUN (mmol/L)	9.00 (5.68, 15.5)	11.7 (6.20, 14.4)	0.976		
ALB (g/L)	29.4 (26.5, 32.5)	29.7 (26.7, 32.5)	0.738		
TP (g/L)	54.6 (49.3, 58.0)	53.3 (50.5, 62.0)	0.818		
ALT (U/L)	24.0 (14.5, 44.5)	45.0 (22.0, 57.0)	0.629		
AST (U/L)	35.0 (22.0, 55.0)	48.0 (37.0, 69.0)	0.538		
ALP (U/L)	141 (104, 202)	143 (92.0, 204)	0.729		
TBIL (μmol/L)	38.3 (15.4, 126)	71.3 (31.0, 162)	0.549		
AKI	21 (67.7%)	1 (100.0%)	0.998		

^*a*^ OR, odds ratio; BMI, body mass index; CKD, chronic kidney disease; ICU, intensive care unit; LRTIs, lower respiratory tract infections; BSIs, bloodstream infections; CRKP, carbapenem-resistant *Klebsiella pneumoniae*; CRPA, carbapenem-resistant *Pseudomonas aeruginosa*; CRAB, carbapenem-resistant *Acinetobacter baumannii*; CRRT, continuous renal replacement therapy; ECMO, extracorporeal membrane oxygenation; CAZ-AVI, ceftazidime/avibactam; BLBLI, beta-lactam-beta-lactamase inhibitor combinations; AUC_ss,24 h_, area under the concentration-to-time curve across 24 h at steady state; CRP; C-reactive protein; PCT, procalcitonin; RBC, red blood cell; WBC, white blood cell; SCr, serum creatinine; BUN, blood urea nitrogen; ALB, serum albumin; TP; total protein; ALT, alanine aminotransferase; AST, aspartate aminotransferase; ALP, alkaline phosphatase; TBIL, total bilirubin; AKI, acute kidney injury. Only surgical removal of infection during polymyxin B treatment was recorded.

**Fig 5:**
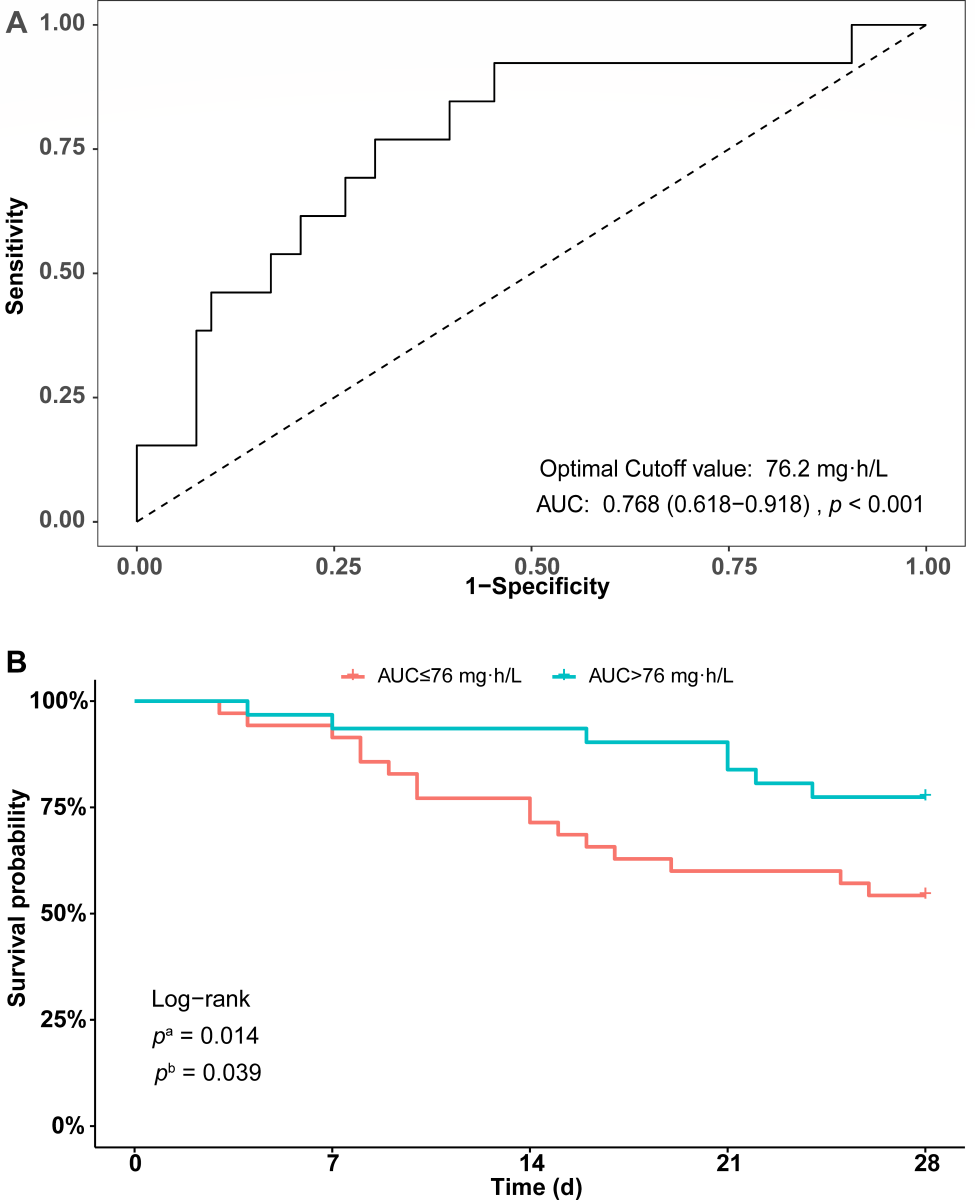
AUC threshold of polymyxin B in IAI patients by ROC analysis (**A**) and survival analysis stratified by the AUC threshold (**B**). *p*^a^ and *p*^b^ indicate significant differences in 14-day and 28-day survival between the two groups, respectively.

We performed ROC analysis for the area under the curve and 14-day mortality of IAI patients, and the sensitivity of the polymyxin B AUC for mortality was 0.768 (*P* < 0.001, Fig. 5A). The optimal cutoff value for the AUC obtained by ROC analysis was 76 mg∙h/L. Subsequent Kaplan–Meier analysis revealed that an AUC over 76 mg∙h/L was associated with much lower 14-day and 28-day mortality (Fig. 5B).

No significant association between the AUC and nephrotoxicity was identified in this study (Table 5).

**TABLE 5:** Univariate and multivariate logistic regression analyses for AKI^*a*^

	Without AKI (*n* = 72)	AKI (*n* = 112)	Univariate logistic regression	Multivariate logistic regression	
			*P*-value	OR (95% CI)	*P*-value
Sex, *n* (%)					
Male	45 (62.5%)	83 (74.1%)	0.096	0.468 (0.228–0.960)	0.038
Female	27 (37.5%)	29 (25.9%)			
Age (years)	68.0 (56.0, 78.0)	70.0 (60.0, 80.0)	0.310		
Weight (kg)	60.0 (55.0, 68.0)	62.3 (53.3, 70.0)	0.615		
BMI (kg/m^2^)	21.6 (19.6, 24.4)	22.4 (19.6, 25.4)	0.573		
Comorbidities, *n* (%)					
Dementia	2 (2.80%)	5 (4.50%)	0.563		
Diabetes	21 (29.2%)	29 (25.9%)	0.626		
CKD	3 (4.20%)	7 (6.30%)	0.546		
Malignancy	18 (25.0%)	34 (30.4%)	0.431		
Heart failure	9 (12.5%)	11 (9.80%)	0.570		
COPD	10 (13.9%)	10 (8.90%)	0.295		
Immunodeficiency	2 (2.80%)	4 (3.60%)	0.768		
Hypertension	35 (48.6%)	41 (36.6%)	0.108	0.446 (0.225–0.884)	0.021
Hyperlipidemia	1 (1.40%)	5 (4.50%)	0.278		
Acute pancreatitis	6 (8.30%)	15 (13.4%)	0.296		
COVID-19	7 (9.70%)	14 (12.5%)	0.564		
ICU admission	62 (86.1%)	100 (89.3%)	0.518		
Severely ill	27 (37.5%)	43 (38.4%)	0.903		
Infected site, *n* (%)					
LRTIs	51 (70.8%)	86 (76.8%)	0.367		
IAIs	10 (13.9%)	22 (19.6%)	0.317		
BSIs	15 (20.8%)	27 (24.1%)	0.606		
Other or undefined	7 (9.70%)	14 (12.5%)	0.564		
Pathogen, *n* (%)					
CRKP	17 (23.6%)	34 (30.4%)	0.320		
CRPA	10 (13.9%)	27 (24.1%)	0.095	3.50 (1.38–8.92)	0.009
CRAB	22 (30.6%)	51 (45.5%)	0.044	3.09 (1.47–6.47)	0.003
Others	38 (52.8%)	46 (41.1%)	0.121		
CRRT					
ECMO	1 (1.40%)	6 (5.40%)	0.202		
Daily dose (mg/d)	100 (100, 150)	150 (100, 150)	0.808		
Dose/weight (mg/kg/12 hours)	1.00 (0.833, 1.36)	1.00 (0.833, 1.24)	0.606		
Aerosol inhalation	10 (13.9%)	13 (11.6%)	0.648		
Concomitant drugs, *n* (%)					
Tigecycline	13 (18.1%)	46 (41.1%)	0.001	4.14 (1.89–9.08)	<0.001
CAZ-AVI	6 (8.30%)	12 (10.7%)	0.597		
Carbapenem	11 (15.3%)	19 (17.0%)	0.763		
BLBLI	28 (38.9%)	36 (32.1%)	0.349		
Aminoglycosides	1 (1.40%)	3 (2.70%)	0.565		
Others	12 (16.7%)	17 (15.2%)	0.787		
None	21 (29.2%)	23 (20.5%)	0.182		
AUC_ss,24 h_ (mg·h/L)	77.2 (57.9, 114)	82.0 (63.9, 113)	0.348		
AUC >100 mg·h/L	50 (69.4%)	74 (66.1%)	0.634		
Laboratory data					
CRP (μg/L)	108 (54.9, 156)	111 (73.8, 161)	0.541		
PCT (ng/mL)	0.870 (0.246, 1.90)	0.810 (0.305, 2.69)	0.955		
RBC (10^12^ /L)	2.63 (2.31, 3.02)	2.65 (2.27, 2.97)	0.826		
WBC (10^9^ /L)	9.41 (6.28, 12.8)	10.1 (6.58, 14.6)	0.261		
Neutrophil (10^9^ /L)	86.5 (77.1, 91.5)	86.5 (75.4, 91.4)	0.966		
SCr (μmol/L)	64.0 (53.7, 98.1)	62.5 (44.0, 92.8)	0.293		
BUN (mmol/L)	8.38 (5.20, 14.2)	9.89 (5.74, 13.6)	0.615		
ALB (g/L)	30.5 (26.6, 31.6)	28.4 (26.0, 31.5)	0.334		
TP (g/L)	56.3 (49.4, 63.2)	54.0 (50.6, 58.8)	0.090		0.322
ALT (U/L)	30.0 (17.3, 56.0)	28.0 (15.0, 53.5)	0.416		
AST (U/L)	35.0 (24.0, 64.5)	34.0 (20.5, 53.5)	0.499		
ALP (U/L)	96.0 (71.5, 195)	110 (78.0, 154)	0.275		
TBIL (μmol/L)	11.1 (7.83, 19.1)	15.0 (9.25, 22.9)	0.652		

^*a*^ OR, odds ratio; BMI, body mass index; CKD, chronic kidney disease; COPD, chronic obstructive pulmonary disease; ICU, intensive care unit; LRTIs, lower respiratory tract infections; IAIs, intra-abdominal infections; BSIs, bloodstream infections; CRKP, carbapenem-resistant *Klebsiella pneumoniae*; CRPA, carbapenem-resistant *Pseudomonas aeruginosa*; CRAB, carbapenem-resistant *Acinetobacter baumannii*; CRRT, continuous renal replacement therapy; ECMO, extracorporeal membrane oxygenation; CAZ-AVI, ceftazidime/avibactam; BLBLI, beta-lactam-beta-lactamase inhibitor combinations; AUC_ss,24 h_, area under the concentration-to-time curve across 24 hours at steady state; CRP; C-reactive protein; PCT, procalcitonin; RBC, red blood cell; WBC, white blood cell; SCr, serum creatinine; BUN, blood urea nitrogen; ALB, serum albumin; TP; total protein; ALT, alanine aminotransferase; AST, aspartate aminotransferase; ALP, alkaline phosphatase; TBIL, total bilirubin.

## DISCUSSION

To the best of our knowledge, this is the largest prospective observational multicenter study to assess the clinical outcomes and PK/PD of intravenous polymyxin B treatment for CRGNB infections with different infection sites. In terms of clinical outcomes, patients with IAI had lower mortality than those with other site infections. The AUC of polymyxin B was significantly associated with mortality for the first time, although only in IAI patients. A new polymyxin B AUC cut-off for IAI patients was provided by this study. The results suggested that the infection site should be considered when dosing and monitoring polymyxin B.

The main strength of this study was the evaluation of PK/PD in different subgroups rather than the combination of patients with different characteristics. Polymyxins have significant infection site-specific PK/PD properties. A preclinical study evaluating the PK/PD relationship in *Klebsiella pneumoniae*-infected mouse models revealed that the *f*AUC/MIC was related to the antibacterial effect on thigh infection, and the target values of the *f*AUC/MIC for stasis and 1 log10 kill were 1.22–13.5 and 3.72–28.0, respectively. However, there was no relationship between the antibacterial effects of polymyxin B and the *f*AUC/MIC, and it was not possible to achieve stasis in lung infection, even at the highest dose tolerated by mice (15). Another study evaluating the PK/PD properties of colistin against PA and AB also reported similar results: for only some strains, it could achieve 2 log-kill in lung infection models at extremely high *f*AUC/MIC values (36.8–105) (20). They attributed the lack of responsiveness of lung infections to relatively low concentrations of polymyxins in the epithelial lining fluid (ELF) of mice that were systemically administered the drug.

However, recent clinical studies have reported confounding results (21, 22). Yang et al. performed a retrospective observational study and reported that an AUC of 50–100 mg∙h/L was associated with decreased nephrotoxicity while ensuring clinical efficacy in critically ill patients. Tang et al. reported that the AUC/MIC was associated with polymyxin B in nosocomial pneumonia patients with CRO, and the cutoff value was 66.9 mg∙h/L when polymyxin B was combined with other antibiotics (23). As we mentioned previously, these two studies included many pneumonia patients who received additional inhaled polymyxin B, which increased the concentration at the infection site and made the plasma concentration less relative (24). Thus, these results need further validation. A subsequent RCT revealed that this target AUC compliance was not correlated with clinical outcomes but with AKI only (25). It should be noted that these studies all used total plasma polymyxin B concentration to calculate the AUC due to the difficulty in determination of the concentration of unbound drugs in routine TDM settings. Our study adequately explained the controversy of previous studies. Clinical PK/PD studies are biased by the inclusion of patients with different infection sites. Our results are in accordance with those of preclinical studies, which revealed that the plasma polymyxin B AUC was related to the clinical outcomes of some site infections other than LRTI. The results indicated that the use of reported AUC targets carefully as the infection site matters.

IAI is the only infection type whose mortality is significantly associated with the AUC of polymyxin B. It may be due to the relatively high distribution in intra-abdominal organs of this drug (26). This is a stronger evidence that supports the TDM of polymyxin B than observed in previous studies. The IAI types in the study were mainly infections after pancreatitis and post-surgery infections. Tube drainage was applied for nearly all patients; thus, we only recorded surgical removal during polymyxin B treatment in this study. Finally, surgical removal was an independent risk factor associated with 14-day mortality, which suggested the importance of proactive source control in IAI patients.

BSI is considered an ideal disease model for PK/PD studies as the plasma concentration directly reflects the drug concentration at the infection site. Our previous study of CRKP BSI patients revealed that an AUC/MIC greater than 54.4 would benefit patients receiving polymyxin B combined with high-dose meropenem therapy (27). Survival analysis revealed that patients with an AUC less than 50 mg∙h/L had increased mortality. However, the association between the AUC and 14-day mortality in patients with BSIs was not significant in this study. The reasons were that the sample size of BSI patients was limited, and most of them had comorbid pneumonia. On the basis of the available evidence, an AUC over 50 mg∙h/L is a reasonable target for BSI patients.

Among all the infection sites, patients with LRTI had poorer clinical outcomes. These results were in accordance with those of previously mentioned preclinical studies and many clinical observations. A study of nosocomial pneumonia caused by MDRPs reported that the favorable clinical outcome was 47.3% in patients receiving intravenous polymyxin B, suggesting that polymyxin B is a reliable drug but can be used only as salvage therapy for nosocomial pneumonia caused by MDRPs (28). A prospective cohort study also revealed that polymyxin B treatment at the currently recommended dosage may result in inferior outcomes compared to other drugs in the treatment of VAP and VAT caused by organisms that are susceptible to this agent *in vitro* (29). Nebulized polymyxin B may be an option for LRTI patients because it can increase the drug concentration at the infection site. The clinical benefit of inhaled polymyxin B is controversial in the current literature (30, 31). There are additional factors that could influence the effect of inhaled polymyxin B (32). In this multicenter study, inhaled polymyxin B did not have beneficial effects. The possible reasons may be that the protocols of inhaled polymyxin B differ among centers and that few patients have received inhaled treatment. Additional evidence is needed.

Combination with other antibiotics is a common strategy for enhancing the bacterial-killing effect and minimizing resistance emergence (33). For example, *in vitro* pharmacodynamics experiments revealed that polymyxin B and tigecycline have synergistic or additive effects on CRAB (34). The combination of antibiotics, namely, ceftazidime-avibactam and amikacin, was found to be an independent risk factor for overall 14-day mortality. Ceftazidime-avibactam is an effective and safe option for KPC-producing CRGNB, and its combined use could reduce the mortality in this study. It would be a confounding factor that makes interpretation of the results challenging. But AUC was not associated with overall mortality by excluding patients receiving ceftazidime-avibactam (Table S11). Polymyxin B combined with amikacin was reported to decrease 30-day mortality in patients with CRKP bloodstream infection (35). However, amikacin was associated with additional death incidences in our study, and the reason was unknown. The effect of combined antibiotics on the PK/PD of polymyxin B should be evaluated in future studies.

CRRT was a risk factor associated with 14-day mortality in both overall patients and IAI patients. Previously, polymyxin B was thought to eliminate mainly the non-kidney pathway, and the dose does not need to be adjusted in patients with CRRT and renal insufficiency (16). However, recent studies reported that CRRT was a covariate in the PPK model and suggested that the dose used was slightly higher (36–38). But the AUCs of the CRRT patients were not lower than those of the other patients in this study. CRRT itself indicates kidney injury, and organ failure is a risk factor for mortality (39). Special attention should be given to these patients to improve their clinical outcomes.

Nephrotoxicity is a well-known dose-dependent toxicity of polymyxin B (40). Previous studies suggested that an AUC of 100 mg∙h/L was the upper limit for polymyxin B exposure (17, 41). In contrast, we did not find a significant difference between patients with AUCs above or below 100 mg∙h/L or the association between AKI and polymyxin B exposure in our study. As in previous studies, we have not analyzed the causality of polymyxin B use and AKI. Many factors in CRGNB-infected patients, such as septic shock, contribute to AKI (42). These factors interfere with the PK/PD analysis of polymyxin B, and future analysis on polymyxin B-induced AKI patients would be helpful. Moreover, the dose range of polymyxin B was narrow, which resulted in a narrow AUC distribution (averages: 77.2 vs 83.0 mg∙h/L; range: 57.9 to 114 mg∙h/L). The percentages of patients having an AUC over 100 mg∙h/L were similar in both groups (Table 4). These issues may contribute to the negative finding in the exposure–nephrotoxicity relationship in this study.

There are several limitations to this study. The sample size for specific infection site patients was insufficient, and that may be the reason that the difference in the mortality among BSI patients with different AUCs was insignificant. The unbound polymyxin B in plasma samples was not determined. The influence of various confounding factors, such as the efficacy of combined antibiotics, needs to be verified in future studies. Polymyxin B MICs of isolated strains were not tested. The upper limit of polymyxin B exposure should be analyzed in those with drug-associated AKI, not all AKI patients.

### Conclusion

We performed a large prospective observational multicenter study of polymyxin B for treating various site CRGNB infections. The clinical outcomes of polymyxin B, as well as nephrotoxicity, were provided. PK/PD analysis indicated that the AUC of polymyxin B was not associated with mortality in LRTI patients but was associated with that in IAI and BSI patients. A new AUC cutoff of polymyxin B was provided for IAI patients. These results would benefit personalized dosing and monitoring of this drug.

## MATERIALS AND METHODS

### Study design and patient enrollment

This study was designed as a prospective, observational, multicenter study and was conducted in accordance with the guidelines of the Declaration of Helsinki. Ethics approval was obtained from the Ethics Committee of Sir Run Run Shaw Hospital, Zhejiang University School of Medicine (reference number 20220124-30). The study was registered in the Chinese Clinical Trial Registry (https://www.chictr.org.cn/) with the number ChiCTR2200056667 (registered 10 February 2022). Patients who met the following criteria were included: (i) identified or highly suspected infection caused by carbapenem-resistant gram-negative bacteria; (ii) received polymyxin B treatment for >72 hours; and (iii) were willing and able to collect plasma samples for concentration determination. The exclusion criteria were as follows: (i) patients aged <18 years; (ii) patients receiving nonintravenous polymyxin B only; and (iii) patients whose peak and trough concentrations were not obtained. Patients who received inhaled CMS or colistin sulfate were excluded because polymyxin B concentration determination was influenced as polymyxin E2 was used as internal standard. However, inhaled polymyxin B does not affect the determination of the plasma concentration, and these patients were included. Written informed consent was obtained from the patients before enrollment according to the institution’s requirements.

### Drug administration, sampling, concentration determination, and AUC calculation

Owing to the observational nature of the study, the dosing of polymyxin B and the regimen of combined antibiotics were not implemented and were decided by doctors. Two blood samples (peak and trough concentrations) were collected after four or more doses of intravenous polymyxin B. Plasma was isolated by centrifugation and stored at −80°C before determination. The polymyxin B concentrations, which were obtained by adding polymyxin B1 (including B1-I) and B2 concentrations, were determined via a previously established LC‒MS/MS method (43). The 24-hour steady-state AUC was calculated via the first-order elimination-based equation method, which is recommended by the Chinese consensus guideline for polymyxin B therapeutic drug monitoring (44).

### Follow-up and clinical outcomes

The patients were followed for up to 28 days, and the clinical evaluation was performed by two researchers. The primary outcomes were 14-day mortality and nephrotoxicity. The secondary outcomes were the clinical response rate and 28-day mortality. Clinical response was defined as the disappearance or improvement in signs and biochemical indicators of infection, which was evaluated at the end of treatment or on the 14th day of treatment if the duration of polymyxin B treatment was longer than 14 days. Nephrotoxicity was evaluated by the occurrence of AKI, which was evaluated based on changes in serum creatinine and the eGFR estimated via the Cockcroft‒Gault equation during polymyxin B treatment (45). The severity of AKI was graded according to the RIFLE criteria (46). Patients who started continuous renal replacement therapy (CRRT) during polymyxin B treatment were defined as having F-AKI. Given that the treatment durations of the enrolled patients were longer than 3 days, all patients were evaluated for mortality and clinical response. Patients receiving CCRT before polymyxin B treatment were excluded from nephrotoxicity evaluation because of the difficulty in evaluating AKI with RIFLE.

### PK/PD and statistical analysis

To explore the association between polymyxin B exposure (AUC) and clinical outcomes, Kaplan–Meier analysis was performed for each outcome by stratifying patients according to the preset AUC threshold. For mortality and clinical response, the AUC threshold was 50 mg∙h/L, whereas for nephrotoxicity, the threshold was 100 mg∙h/L. Subgroup analysis was carried out based on the infection site, including LRTI, intra-abdominal infection (IAI), and bloodstream infection (BSI). Data are presented as the means and standard errors or medians and interquartile ranges for continuous variables and as frequencies (percentages) for categorical variables. Student’s t test and the Mann‒Whitney test were used for comparison of normally and nonnormally distributed continuous variables, respectively. The χ test or Fisher’s exact test was used for comparison of categorical variables. Multivariate logistic regression was used to identify any independent variables associated with primary and secondary outcomes, which included variables with *P* < 0.2 in the univariate analysis. If the AUC was an independent variable, ROC analysis was performed to explore a new threshold of the AUC, and further Kaplan–Meier analysis was performed. *P* < 0.05 was considered statistically significant.

## Data Availability

The data sets used and analyzed during the current study are available from the corresponding author on reasonable request.

## SUPPLEMENTAL MATERIAL

Tables S1 to S11 aac.01859-24-s0001.docx Multivariate logistic analysis for secondary outcomes and other infection types.

ASM does not own the copyrights to Supplemental Material that may be linked to, or accessed through, an article. The authors have granted ASM a non-exclusive, world-wide license to publish the Supplemental Material files. Please contact the corresponding author directly for reuse.
