# Supplementary material for: Clinical outcomes and pharmacokinetics/pharmacodynamics of intravenous polymyxin B treatment for various site carbapenem-resistant gram-negative bacterial infections: a prospective observational multicenter study
Source: Antimicrob Agents Chemother. 2025 Mar 6;69(4):e01859-24. doi: 10.1128/aac.01859-24 (PMC11963601; doi:10.1128/aac.01859-24)
Supplement: Tables S1 to S11 — Multivariate logistic analysis for secondary outcomes and other infection types. [file aac.01859-24-s0001.docx]

Table S1 Univariate and multivariate logistic regression analyses for 14-day mortality of pulmonary infection patients.

|  | Survival | No survival | Univariate logistic regression | Multivariate logistic regression | |
| --- | --- | --- | --- | --- | --- |
|  | (n=162) | (n=77) | p-value | OR (95% CI) | p-value |
| Age (years) | 70.0 (59.3, 80.8) | 76.0 (64.0, 84.0) | 0.091 |  | 0.785 |
| Diabetes | 41 (25.3%) | 28 (36.4%) | 0.079 |  | 0.356 |
| Immunodeficiency | 15 (9.3%) | 3 (3.9%) | 0.154 | 0.213 (0.053-0.855) | 0.029 |
| Covid19 | 13 (8.0%) | 21 (27.3%) | 0.001 | 4.302 (1.81-10.222) | 0.001 |
| CRKP | 43 (26.5%) | 12 (15.6%) | 0.063 |  | 0.245 |
| CRAB | 80 (49.4%) | 48 (62.3%) | 0.062 |  | 0.292 |
| CRRT | 61 (37.7%) | 41 (53.2%) | 0.024 | 2.545 (1.271-5.099) | 0.008 |
| CAZ-AVI | 26 (16.0%) | 6 (7.8%) | 0.086 | 0.171 (0.048-0.605) | 0.006 |
| WBC (10^9^/L) | 10.0 (6.12, 14.0) | 11.4 (6.70, 16.7) | 0.081 |  | 0.139 |
| SCr (μmol/L) | 69.0 (51.5, 108) | 97.5 (62.0, 148) | 0.028 | 1.004 (1-1.007) | 0.028 |
| ALB (g/L) | 29.5 (27.3, 32.3) | 28.2 (25.7, 30.7) | 0.012 |  | 0.238 |
| ALP (U/L) | 113 (79.0, 170) | 102 (64.0, 144) | 0.123 |  | 0.265 |
| AKI | 65 (40.1%) | 21 (27.3%) | 0.031 |  | 0.863 |

Note: OR, odd ratio; CRKP, carbapenem-resistant *klebsiella pneumoniae*; CRAB, carbapenem-resistant *acinetobacter baumannii*; CRRT, continuous renal replacement therapy; CAZ-AVI, ceftazidime / avibactam; WBC, white blood cell; SCr, serum creatinine; ALB, serum albumin; ALP, alkaline phosphatase; AKI, acute kidney injury.

Table S2 Univariate and multivariate logistic regression analyses for 14-day mortality of blood stream infection patients.

|  | Survival | No survival | Univariate logistic regression | Multivariate logistic regression |  |
| --- | --- | --- | --- | --- | --- |
|  | (n=53) | (n=23) | p-value | OR (95% CI) | p-value |
| Covid19 | 4 (7.5%) | 5 (21.7%) | 0.091 |  | 0.109 |
| CRKP | 24 (45.3%) | 3 (13.0%) | 0.012 |  | 0.173 |
| CRRT | 18 (34.0%) | 16 (69.6%) | 0.006 | 7.596 (1.776-32.482) | 0.006 |
| Aerosol inhalation | 13 (24.5%) | 1 (4.3%) | 0.066 |  | 0.192 |
| WBC | 9.75 (3.85, 14.2) | 11.4 (8.00, 20.4) | 0.099 |  | 0.852 |
| SCr (μmol/L) | 83.0 (60.0, 117) | 103 (72.0, 210) | 0.046 |  | 0.570 |
| BUN | 11.9 (5.97, 17.9) | 17.4 (9.89, 34.2) | 0.006 |  | 0.351 |
| ALB (g/L) | 29.8 (27.1, 33.1) | 26.9 (24.2, 30.2) | 0.005 | 0.706 (0.564-0.882) | 0.002 |
| AKI | 22 (41.5%) | 6 (26.1%) | 0.012 |  | 0.363 |

Note: OR, odd ratio; CRKP, carbapenem-resistant *klebsiella pneumoniae*; CRRT, continuous renal replacement therapy; WBC, white blood cell; SCr, serum creatinine; BUN, blood urea nitrogen; ALB, serum albumin; AKI, acute kidney injury.

Table S3 Univariate and multivariate logistic regression analyses for clinical response.

|  | Failure | Response | Univariate logistic regression | Multivariate logistic regression | |
| --- | --- | --- | --- | --- | --- |
|  | (n=168) | (n=144) | p-value | OR (95% CI) | p-value |
| Malignancy | 50 (29.8%) | 25 (17.4%) | 0.011 | 0.426 (0.225-0.805) | 0.009 |
| Covid 19 | 26 (15.5%) | 12 (8.3%) | 0.058 |  | 0.175 |
| CRAB | 85 (50.6%) | 56 (38.9%) | 0.039 |  | 0.135 |
| CRRT | 83 (49.4%) | 45 (31.3%) | 0.002 | 0.406 (0.226-0.727) | 0.002 |
| Tigecycline | 62 (36.9%) | 41 (28.5%) | 0.115 |  | 0.893 |
| Carbapenem | 37 (22.0%) | 23 (16.0%) | 0.178 |  | 0.200 |
| Aminoglycosides | 9 (5.4%) | 1 (0.7%) | 0.049 | 0.100 (0.012-0.859) | 0.036 |
| RBC (10^12^/L) | 2.44 (2.14, 2.87) | 2.60 (2.21, 3.01) | 0.034 |  | 0.164 |
| BUN (mmol/L) | 11.6 (7.28, 19.1) | 10.5 (6.28, 15.5) | 0.096 |  | 0.729 |
| ALB (g/L) | 28.2 (25.9, 30.8) | 30.4 (27.5, 32.9) | 0.001 | 1.147 (1.074-1.226) | <0.001 |
| TBIL (μmol/L) | 18.4 (10.2, 61.8) | 14.9 (8.90, 27.6) | 0.005 | 0.995 (0.99-0.999) | 0.020 |
| AKI | 55 (32.7%) | 57 (39.6%) | <0.001 |  | 0.640 |

Note: OR, odd ratio; CRAB, carbapenem-resistant *acinetobacter baumannii*; CRRT, continuous renal replacement therapy; RBC, red blood cell; BUN, blood urea nitrogen; ALB, serum albumin; TBIL, total bilirubin; AKI, acute kidney injury.

Table S4 Univariate and multivariate logistic regression analyses for 28-day mortality.

|  | Survival | No survival | Univariate logistic regression | Multivariate logistic regression | |
| --- | --- | --- | --- | --- | --- |
|  | (n=193) | (n=119) | p-value | OR (95% CI) | p-value |
| Age (years) | 68.0 (57.0, 78.0) | 73.0 (59.5, 82.0) | 0.092 |  | 0.570 |
| Heart failure | 20 (10.4%) | 20 (16.8%) | 0.101 |  | 0.318 |
| Immunodeficiency | 16 (8.30%) | 4 (3.40%) | 0.095 |  | 0.088 |
| Acute pancreatitis | 28 (14.5%) | 9 (7.60%) | 0.070 | 0.298 (0.118-0.754) | 0.011 |
| Covid19 | 15 (7.80%) | 23 (19.3%) | 0.003 | 3.298 (1.416-7.682) | 0.006 |
| ICU admission | 172 (89.1%) | 118 (99.2%) | 0.010 |  | 0.146 |
| CRKP | 61 (31.6%) | 25 (21.0%) | 0.043 |  | 0.604 |
| CRAB | 77 (39.9%) | 64 (53.8%) | 0.017 |  | 0.200 |
| CRRT | 60 (31.1%) | 68 (57.1%) | 0.001 | 2.510 (1.370-4.600) | 0.003 |
| Daily dose (mg/d) | 150 (100, 150) | 150 (100, 150) | 0.098 | 1.011 (1.000-1.023) | 0.046 |
| Aminoglycosides | 2 (1.00%) | 8 (6.70%) | 0.016 | 8.557 (1.581-46.304) | 0.013 |
| WBC (10^9^/L) | 9.51 (5.48, 13.6) | 11.4 (6.61, 17.3) | 0.007 |  | 0.103 |
| BUN (mmol/L) | 9.12 (5.75, 14.8) | 13.6 (8.49, 21.1) | 0.001 | 1.033 (1.004-1.064) | 0.028 |
| ALB (g/L) | 30.0 (27.3, 32.0) | 27.9 (25.8, 30.7) | 0.001 | 0.918 (0.855-0.986) | 0.018 |
| TBIL (μmol/L) | 15.1 (9.00, 31.8) | 19.0 (10.1, 56.3) | 0.049 |  | 0.150 |
| AKI | 81 (42.0%) | 31 (26.1%) | <0.001 |  | 0.898 |

Note: OR, odd ratio; CRKP, carbapenem-resistant *klebsiella pneumoniae*; CRAB, carbapenem-resistant *acinetobacter baumannii*; CRRT, continuous renal replacement therapy; WBC, white blood cell; BUN, blood urea nitrogen; ALB, serum albumin; TBIL, total bilirubin; AKI, acute kidney injury.

Table S5 Univariate and multivariate logistic regression analyses for clinical response of pulmonary infection patients.

|  | Failure | Response | Univariate logistic regression | Multivariate logistic regression | |
| --- | --- | --- | --- | --- | --- |
|  | (N=133) | (N=106) | p-value | OR (95% CI) | p-value |
| Malignancy | 37 (27.8%) | 16 (15.1%) | 0.02 | 0.434 (0.188-1.001) | 0.050 |
| Immunodeficiency | 6 (4.5%) | 12 (11.3%) | 0.055 |  | 0.188 |
| Covid19 | 25 (18.8%) | 9 (8.5%) | 0.027 |  | 0.066 |
| Severely ill | 80 (60.2%) | 52 (49.1%) | 0.087 |  | 0.535 |
| CRRT | 69 (51.9%) | 33 (31.1%) | 0.001 | 0.219 (0.1-0.48) | <0.001 |
| Tigecycline | 46 (34.6%) | 27 (25.5%) | 0.130 |  | 0.480 |
| Carbapenem | 29 (21.8%) | 13 (12.3%) | 0.057 |  | 0.131 |
| Aminoglycosides | 7 (5.3%) | 1 (0.9%) | 0.102 |  | 0.059 |
| PCT (ng/mL) | 1.40 (0.520, 4.69) | 0.970 (0.303, 3.35) | 0.168 | 1.043 (1.006-1.082) | 0.024 |
| RBC (10^12^/L) | 2.49 (2.20, 2.89) | 2.66 (2.26, 3.01) | 0.083 |  | 0.163 |
| BUN (mmol/L) | 12.7 (8.21, 19.3) | 10.6 (6.27, 15.5) | 0.056 |  | 0.410 |
| ALB (g/L) | 28.3 (26.0, 30.7) | 30.9 (27.5, 33.6) | 0.001 | 1.169 (1.071-1.275) | <0.001 |
| TBIL (μmol/L) | 18.3 (9.30, 58.9) | 14.3 (8.95, 23.1) | 0.012 | 0.993 (0.987-0.999) | 0.032 |
| AKI | 41 (30.8%) | 45 (42.5%) | 0.001 |  | 0.470 |

Note: OR, odd ratio; CRRT, continuous renal replacement therapy; PCT, procalcitonin; RBC, red blood cell; BUN, blood urea nitrogen; ALB, serum albumin; TBIL, total bilirubin; AKI, acute kidney injury.

Table S6 Univariate and multivariate logistic regression analyses for 28-day mortality of pulmonary infection patients.

|  | Survival | No survival | Univariate logistic regression | Multivariate logistic regression | |
| --- | --- | --- | --- | --- | --- |
|  | (n=140) | (n=99) | p-value | OR (95% CI) | p-value |
| Malignancy | 25 (17.9%) | 28 (28.3%) | 0.058 | 2.515 (1.171-5.402) | 0.018 |
| Heart failure | 17 (12.1%) | 20 (20.2%) | 0.093 | 2.638 (1.1-6.326) | 0.030 |
| Immunodeficiency | 14 (10.0%) | 4 (4.0%) | 0.096 |  | 0.282 |
| Covid19 | 11 (7.9%) | 23 (23.2%) | 0.001 | 3.551 (1.408-8.955) | 0.007 |
| Severely ill | 70 (50.0%) | 62 (62.6%) | 0.054 |  | 0.920 |
| CRKP | 37 (26.4%) | 18 (18.2%) | 0.138 |  | 0.680 |
| CRPA | 35 (25.0%) | 17 (17.2%) | 0.15 |  | 0.306 |
| CRRT | 47 (33.6%) | 55 (55.6%) | 0.001 | 3.31 (1.703-6.432) | <0.001 |
| Daily dose (mg/d) | 100 (100, 150) | 150 (100, 150) | 0.098 |  | 0.355 |
| Aminoglycosides | 1 (0.7%) | 7 (7.1%) | 0.029 | 32.018 (2.977-344.381) | 0.004 |
| WBC (10^9^/L) | 9.71 (6.06, 13.4) | 11.2 (6.61, 17.0) | 0.015 | 1.067 (1.022-1.115) | 0.003 |
| SCr (μmol/L) | 66.5 (48.0, 108) | 91.0 (59.5, 142) | 0.117 |  | 0.263 |
| ALB (g/L) | 29.9 (27.6, 32.3) | 27.9 (25.8, 30.7) | 0.001 | 0.89 (0.82-0.965) | 0.005 |
| ALP (U/L) | 113 (79.0, 170) | 104 (71.0, 150) | 0.157 | 0.996 (0.993-1) | 0.046 |
| TBIL (μmol/L) | 15.3 (9.00, 29.2) | 16.1 (9.45, 40.0) | 0.189 |  | 0.167 |
| AKI | 59 (42.1%) | 27 (27.3%) | 0.001 |  | 0.571 |

Note: OR, odd ratio; BMI, body mass index; CRKP, carbapenem-resistant *klebsiella pneumoniae*; CRPA, carbapenem-resistant *pseudomonas aeruginosa*; CRRT, continuous renal replacement therapy; WBC, white blood cell; SCr, serum creatinine; ALB, serum albumin; ALP, alkaline phosphatase; TBIL, total bilirubin; TBIL, total bilirubin; AKI, acute kidney injury.

Table S7 Univariate and multivariate logistic regression analysesfor clinical response of intra-abdominal infection patients.

|  | Failure | Response | Univariate logistic regression | Multivariate logistic regression | |
| --- | --- | --- | --- | --- | --- |
|  | (N=37) | (N=29) | p-value | OR (95% CI) | p-value |
| Covid19 | 1 (2.7%) | 4 (13.8%) | 0.127 |  | 0.520 |
| CRPA | 11 (29.7%) | 4 (13.8%) | 0.133 | 0.164 (0.035-0.762) | 0.021 |
| CRAB | 23 (62.2%) | 7 (24.1%) | 0.003 | 0.101 (0.027-0.376) | 0.001 |
| CRRT | 24 (64.9%) | 10 (34.5%) | 0.016 |  | 0.132 |
| Daily dose (mg/d) | 150 (150, 150) | 150 (100, 150) | 0.069 | 0.972 (0.946-0.998) | 0.037 |
| Carbapenem | 12 (32.4%) | 5 (17.2%) | 0.167 |  | 0.179 |
| AUC_ss,24 h_ (mg·h/L) | 67.7 (50.5, 100) | 78.0 (62.8, 94.9) | 0.129 |  | 0.303 |
| Neutrophil (10^9^/L) | 89.2 (83.4, 93.2) | 84.6 (80.5, 90.4) | 0.139 |  | 0.140 |
| AKI | 10 (27.0%) | 12 (41.4%) | 0.013 |  | 0.912 |

Note: OR, odd ratio; CRPA, carbapenem-resistant *pseudomonas aeruginosa*; CRAB, carbapenem-resistant *acinetobacter baumannii*; CRRT, continuous renal replacement therapy; AUC_ss,24 h_, area under the concentration-to-time curve across 24 h at steady state; AKI, acute kidney injury.

Table S8 Univariate and multivariate logistic regression analyses for 28-day mortality of intra-abdominal infection patients.

|  | Survival | No survival | Univariate logistic regression | Multivariate logistic regression | |
| --- | --- | --- | --- | --- | --- |
|  | (n=43) | (n=23) | p-value | OR (95% CI) | p-value |
| Diabetes | 5 (11.6%) | 3 (13.0%) | 0.015 |  | 0.755 |
| Acute pancreatitis | 25 (58.1%) | 6 (26.1%) | 0.016 | 0.169 (0.041-0.688) | 0.013 |
| CRPA | 7 (16.3%) | 8 (34.8%) | 0.094 |  | 0.354 |
| CRRT | 16 (37.2%) | 18 (78.3%) | 0.002 | 4.647 (1.237-17.458) | 0.023 |
| Daily dose (mg/d) | 150 (100, 150) | 150 (150, 150) | 0.078 | 1.037 (1.001-1.074) | 0.042 |
| Carbapenem | 8 (18.6%) | 9 (39.1%) | 0.074 |  | 0.484 |
| AUC_ss,24 h_ (mg·h/L) | 77.2 (66.0, 104) | 59.4 (46.0, 81.8) | 0.042 | 0.975 (0.953-0.997) | 0.024 |
| TBIL | 23.4 (14.8, 95.0) | 89.3 (44.2, 184) | 0.047 |  | 0.249 |
| AKI | 19 (44.2%) | 3 (13.0%) | 0.003 |  | 0.484 |

Note: OR, odd ratio; CRPA, carbapenem-resistant *pseudomonas aeruginosa*; CRAB, carbapenem-resistant *acinetobacter baumannii*; CRRT, continuous renal replacement therapy; AUC_ss,24 h_, area under the concentration-to-time curve across 24 h at steady state; TBIL, total bilirubin; AKI, acute kidney injury.

Table S9 Univariate and multivariate logistic regression analyses for clinical response of blood stream infection patients.

|  | Failure | Response | Univariate logistic regression | Multivariate logistic regression | |
| --- | --- | --- | --- | --- | --- |
|  | (N=41) | (N=35) | p-value | OR (95% CI) | p-value |
| CKD | 2 (4.9%) | 7 (20.0%) | 0.059 | 9.998 (1.257-79.497) | 0.030 |
| Hypertension | 16 (39.0%) | 20 (57.1%) | 0.117 | 3.909 (1.149-13.293) | 0.029 |
| Covid19 | 7 (17.1%) | 2 (5.7%) | 0.145 | 0.132 (0.018-0.963) | 0.046 |
| CRRT | 22 (53.7%) | 12 (34.3%) | 0.093 |  | 0.057 |
| BLBLI | 15 (36.6%) | 5 (14.3%) | 0.033 | 0.143 (0.029-0.697) | 0.016 |
| AUC≥50 mg·h/L | 34 (82.9%) | 33 (94.3%) | 0.145 |  | 0.708 |
| ALB (g/L) | 29.1 (25.7, 30.8) | 29.8 (27.3, 33.5) | 0.035 | 1.127 (0.992-1.281) | 0.066 |
| TBIL (μmol/L) | 18.7 (10.0, 92.9) | 15.0 (7.80, 34.9) | 0.156 |  | 0.299 |
| AKI | 14 (34.1%) | 14 (40.0%) | 0.122 |  | 0.556 |

Note: OR, odd ratio; CKD, chronic kidney disease; CRRT, continuous renal replacement therapy; BLBLI, beta-lactam-beta-lactamase inhibitor combinations; AUC, area under the concentration-to-time curve across 24 h at steady state; ALB, serum albumin; TBIL, total bilirubin; AKI, acute kidney injury.

Table S10 Univariate and multivariate logistic regression analyses for 28-day mortality of blood stream infection patients.

|  | Survival | No survival | Univariate logistic regression | Multivariate logistic regression | |
| --- | --- | --- | --- | --- | --- |
|  | (n=45) | (n=31) | p-value | OR (95% CI) | p-value |
| Diabetes | 18 (40.0%) | 6 (19.4%) | 0.062 |  | 0.058 |
| CRRT | 12 (26.7%) | 22 (71.0%) | 0.001 | 7.779 (1.504-40.23) | 0.014 |
| BLBLI | 9 (20.0%) | 11 (35.5%) | 0.136 |  | 0.595 |
| AUC≥50 mg·h/L | 43 (95.6%) | 24 (77.4%) | 0.029 |  | 0.490 |
| SCr (μmol/L) | 80.0 (58.0, 117) | 97.5 (74.8, 181) | 0.074 |  | 0.100 |
| BUN (mmol/L) | 8.94 (5.23, 16.4) | 17.4 (11.1, 24.7) | 0.005 |  | 0.171 |
| ALB (g/L) | 30.0 (27.2, 33.3) | 27.9 (25.3, 30.5) | 0.012 | 0.721 (0.561-0.927) | 0.011 |
| TBIL (μmol/L) | 12.5 (7.80, 37.9) | 36.6 (11.7, 69.8) | 0.143 |  | 0.156 |
| AKI | 22 (48.9%) | 6 (19.4%) | 0.001 |  | 0.432 |

Note: OR, odd ratio; CRRT, continuous renal replacement therapy; BLBLI, beta-lactam-beta-lactamase inhibitor combinations; AUC, area under the concentration-to-time curve across 24 h at steady state; SCr, serum creatinine; BUN, blood urea nitrogen; ALB, serum albumin; TBIL, total bilirubin; AKI, acute kidney injury.

Table S11 Univariate and multivariate logistic regression analyses for 14-day mortality of patients excluding those receiving ceftazidime/avibactam.

|  | Survival  (n=184) | No survival  (n=85) | Univariate logistic regression | Multivariate logistic regression | |
| --- | --- | --- | --- | --- | --- |
|  |  |  | p-value | OR (95% CI) | p-value |
| Sex, n (%) |  |  |  |  |  |
| Male | 131 (71.2%) | 61 (71.8%) | 0.923 |  |  |
| Female | 53 (28.8%) | 24 (28.2%) |  |  |  |
| Age (years) | 69.0 (58.8, 79.3) | 74.0 (61.0, 82.0) | 0.191 |  | 0.926 |
| Weight (kg) | 60.8 (55.0, 70.0) | 63.0 (52.1, 70.0) | 0.263 |  |  |
| BMI (kg/m2) | 22.0 (20.0, 24.6) | 22.8 (19.6, 25.8) | 0.281 |  |  |
| Comorbidities, n (%) |  |  |  |  |  |
| Dementia | 7 (3.80%) | 4 (4.70%) | 0.729 |  |  |
| Diabetes | 46 (25.0%) | 31 (36.5%) | 0.054 |  | 0.439 |
| CKD | 19 (10.3%) | 10 (11.8%) | 0.724 |  |  |
| Malignancy | 44 (23.9%) | 17 (20.0%) | 0.477 |  |  |
| Heart failure | 24 (13.0%) | 13 (15.3%) | 0.619 |  |  |
| COPD | 20 (10.9%) | 6 (7.10%) | 0.329 |  |  |
| Immunodeficiency | 16 (8.70%) | 2 (2.40%) | 0.071 |  | 0.093 |
| Hypertension | 88 (47.8%) | 41 (48.2%) | 0.950 |  |  |
| Hyperlipidemia | 6 (3.30%) | 3 (3.50%) | 0.909 |  |  |
| Acute pancreatitis | 23 (12.5%) | 6 (7.10%) | 0.187 |  |  |
| COVID 19 | 14 (7.60%) | 18 (21.2%) | 0.002 | 4.02 (1.66-9.71) | 0.002 |
| ICU admission | 171 (92.9%) | 84 (98.8%) | 0.076 |  | 0.535 |
| Severely ill | 101 (54.9%) | 55 (64.7%) | 0.130 |  | 0.583 |
| Surgical removal of infection | 26 (14.1%) | 7 (8.20%) | 0.099 |  |  |
| Infected site, n (%) |  |  |  |  |  |
| LRTIs | 136 (73.9%) | 71 (83.5%) | 0.084 |  |  |
| IAIs | 43 (23.4%) | 12 (14.1%) | 0.083 |  | 0.062 |
| BSIs | 43 (23.4%) | 20 (23.5%) | 0.977 |  |  |
| Other or undefined | 20 (10.9%) | 11 (12.9%) | 0.621 |  |  |
| Pathogen, n (%) |  |  |  |  |  |
| CRKP | 55 (29.9%) | 16 (18.8%) | 0.058 |  | 0.628 |
| CRPA | 41 (22.3%) | 16 (18.8%) | 0.519 |  |  |
| CRAB | 74 (40.2%) | 47 (55.3%) | 0.021 |  | 0.054 |
| others | 87 (47.3%) | 31 (36.5%) | 0.098 |  | 0.183 |
| CRRT | 58 (31.5%) | 45 (52.9%) | p<0.001 | 3.20 (1.62-6.31) | p<0.001 |
| ECMO | 13 (7.10%) | 5 (5.90%) | 0.719 |  |  |
| Daily dose (mg/d) | 150 (100, 150) | 150 (100, 150) | 0.820 |  |  |
| dose/weight (mg/kg/12h) | 1.04 (0.833, 1.25) | 1.06 (0.788, 1.26) | 0.851 |  |  |
| Aerosol inhalation | 26 (14.1%) | 10 (11.8%) | 0.597 |  |  |
| Concomitant drugs, n (%) |  |  |  |  |  |
| Tigecycline | 55 (29.9%) | 29 (34.1%) | 0.487 |  |  |
| Carbapenem | 38 (20.7%) | 16 (18.8%) | 0.728 |  |  |
| BLBLI | 62 (33.7%) | 32 (37.6%) | 0.528 |  |  |
| Aminoglycosides | 3 (1.60%) | 5 (5.90%) | 0.074 | 7.95 (1.30-48.5) | 0.025 |
| Other | 35 (19.0%) | 8 (9.40%) | 0.050 |  | 0.183 |
| None | 51 (27.7%) | 18 (21.2%) | 0.255 |  |  |
| AUCss,24 h (mg·h/L) | 76.6 (58.9, 109) | 76.3 (52.2, 103) | 0.982 |  |  |
| Laboratory data |  |  |  |  |  |
| CRP (μg/L) | 123 (70.6, 185) | 125 (70.4, 175) | 0.613 |  |  |
| PCT (ng/mL) | 1.21 (0.400, 5.56) | 1.63 (0.585, 4.47) | 0.291 |  |  |
| RBC (1012/L) | 2.54 (2.19, 2.96) | 2.48 (2.16, 2.90) | 0.454 |  |  |
| WBC (109/L) | 9.60 (5.90, 14.0) | 11.5 (7.50, 17.2) | 0.008 | 1.04 (1.00-1.09) | 0.039 |
| Neutrophil (109/L) | 86.7 (77.6, 91.8) | 89.3 (82.6, 92.6) | 0.097 |  |  |
| SCr (μmol/L) | 70.5 (53.0, 111) | 91.0 (59.0, 144) | 0.120 |  | 0.480 |
| BUN (mmol/L) | 9.90 (6.26, 15.5) | 13.6 (8.37, 21.2) | 0.002 |  | 0.119 |
| ALB (g/L) | 29.6 (27.1, 32.4) | 28.0 (25.5, 30.8) | 0.004 |  | 0.163 |
| TP (g/L) | 55.7 (50.8, 60.7) | 53.3 (47.7, 58.6) | 0.036 |  | 0.737 |
| ALT (U/L) | 28.0 (15.0, 60.0) | 31.0 (15.5, 60.5) | 0.534 |  |  |
| AST (U/L) | 33.0 (22.0, 63.5) | 41.0 (30.0, 72.8) | 0.727 |  |  |
| ALP (U/L) | 115 (80.0, 175) | 104 (68.0, 146) | 0.178 |  | 0.130 |
| TBIL (μmol/L) | 15.1 (9.65, 34.6) | 17.4 (8.70, 37.3) | 0.350 |  |  |
| AKI | 76 (41.3%) | 24 (28.2%) | 0.972 |  |  |

Note: OR, odd ratio; BMI, body mass index; CKD, chronic kidney disease; COPD, chronic obstructive pulmonary disease; ICU, intensive care unit; LRTIs, lower respiratory tract infections; IAIs, intra-abdominal infections; BSIs, blood stream infections; CRKP, carbapenem-resistant *klebsiella pneumoniae*; CRPA, carbapenem-resistant *pseudomonas aeruginosa*; CRAB, carbapenem-resistant *acinetobacter baumannii*; CRRT, continuous renal replacement therapy; ECMO, extracorporeal membrane oxygenation; CAZ-AVI, ceftazidime / avibactam; BLBLI, beta-lactam-beta-lactamase inhibitor combinations; AUC_ss,24 h_, area under the concentration-to-time curve across 24 h at steady state; CRP; C-reactive protein; PCT, procalcitonin; RBC, red blood cell; WBC, white blood cell; SCr, serum creatinine; BUN, blood urea nitrogen; ALB, serum albumin; TP; total protein; ALT, alanine aminotransferase; AST, aspartate aminotransferase; ALP, alkaline phosphatase; TBIL, total bilirubin; AKI, acute kidney injury.
